# Key Amino Acid Residues Involved in Binding Interactions between *Bactrocera minax* Odorant-Binding Protein 3 (BminOBP3) and Undecanol

**DOI:** 10.3390/insects14090745

**Published:** 2023-09-05

**Authors:** Ling Yang, Xiaoli Tian, Lianyou Gui, Fulian Wang, Guohui Zhang

**Affiliations:** 1 College of Agriculture, Yangtze University, Jingzhou 434025, China; 202071666@yangtzeu.edu.cn (L.Y.); guilianyou@126.com (L.G.); wangfl_hb@163.com (F.W.); 2 College of Life Science, Yangtze University, Jingzhou 434025, China; lyacxiaoli@163.com

**Keywords:** odorant binding protein, site-directed mutagenesis, C-terminus deletion

## Abstract

**Simple Summary:**

Odorant-binding proteins (OBPs) play a crucial role in insect olfaction perception in which OBPs are believed to discriminate, capture, and ferry odors to olfactory receptors, and thus, OBP-based behavioral interference is thought to be a potential novel pest management measure. Our previous study demonstrated that *Bactrocera minax* OBP3 (BminOBP3) exhibited strong binding affinity only with undecanol, which is believed to be a main *B. minax* sex pheromone, among 13 ligands tested. Here, homology modeling and molecular docking were carried out to explore the interaction mechanism between BminOBP3 and undecanol. The results suggested that the C-terminus (I116-P122), especially the last three amino acids of the C-terminus (V120-P122), play an essential role in the ligand binding for BminOBP3, and this was further proven by mutagenesis studies and fluorescence binding assays. These findings have provided important insights into the molecular mechanism by which BminOBP3 interacts with undecanol. Moreover, this study could serve as a significant reference for developing potentially useful technology to control pests, although disturbing their olfaction judgments.

**Abstract:**

Insect odorant-binding proteins (OBPs) are significant in binding and transporting odorants to specific receptors. Our previous study demonstrated that BminOBP3 exhibited a strong affinity with undecanol. However, the binding mechanism between them remains unknown. Here, using homology modeling and molecular docking, we found that the C-terminus (I116-P122), especially the hydrogenbonds formed by the last three amino acid residues (V120, F121, and P122) of the C-terminus, is essential for BminOBP3′s ligand binding. Mutant binding assays showed that the mutant T-OBP3 that lacks C-terminus (I116-P122) displayed a significant decrease in affinity to undecanol (K_i_ = 19.57 ± 0.45) compared with that of the wild-type protein BminOBP3 (K_i_ = 11.59 ± 0.51). In the mutant 3D2a that lacks F121 and P122 and the mutant V120A in which V120 was replaced by alanine, the bindings to undecanol were completely abolished. In conclusion, the C-terminus plays a crucial role in the binding interactions between BminOBP3 and undecanol. Based on the results, we discussed the ligand-binding process of BminOBP3.

## 1. Introduction

Olfaction, the sense of smell, is used by insects to detect and interpret crucial information from volatile semiochemicals in the environment, which drives their vital behaviors, such as searching for food sources, finding mating partners, and escaping from natural predators or enemies. Insect OBPs are small water-soluble carrier proteins concentrated in the lymph of chemosensilla that are distributed on the surface of maxillary palps and antennae (just like ‘noses’ in mammals) [1,2]. Biochemical data suggest that OBPs can selectively bind and ferry external semiochemicals across the aqueous sensillar lymph to olfactory receptors (ORs), which are expressed on the dendritic membranes of olfactory sensory neurons (OSNs) in olfactory sensilla [3,4,5]. Upon semiochemicals binding with ORs, ORs change the chemical stimuli to nervous impulses, activating signal transduction pathways, and ultimately lead to corresponding olfactory behaviors [6,7,8]. Accumulated studies have shown that the disturbance in OBP expression induces a significant impairment in the olfactory function of insects. Xu et al. showed that the *Drosophila* OBP76a (LUSH) mutant is completely devoid of sensitivity to the *Drosophila* pheromone (11-*cis* vaccenyl acetate, cVA) [9]. Silencing in *Culex quinquefasciatus* of *CquiOBP1* induces asignificant decline of electrophysiological responses to oviposition attractants [10]. The repellent behavior of *Acyrthosiphon pisum* to the sesquiterpene *E*-*β*-farnesene (EBF) disappears after knocking down *ApisOBP3* and *ApisOBP7* [11]. The behavioral response of *Bactrocera dorsalis* to methyl eugenol significantly decreased when *BdorOBP2*was silenced [12].

Although extensive research has proven that OBPs are indispensable in insects’ odorant recognition, the interaction mechanism between insect OBPs and odors remains largely unknown. Structural studies on OBP–ligand complexes have shed some light on the ligand-binding mechanism. Structural analysis of *Bombyx mori* pheromone-binding protein (BmorPBP) bound with bombykol indicated that bombykol is bound in BmorPBP’s cavity vianumerous hydrophobic interactions [13] and the hydrogen bond interaction between bombykol and Ser56 in BmorPBP’s cavity [14]. However, when bombykol is bound to *Bombyx mori* general odorant-binding protein 2 (BmorGOBP2), the hydrogen bond is established with Arg110 rather than with Ser56 as found for BmorPBP [15]. Thr57 in the binding pocket of *Drosophila melanogaster* OBP LUSH forms a hydrogen bond with alcohol, which has proven to be a key force in LUSH’s binding to alcohols [16]. In *Loxostege sticticalis*, Thr15 and Trp43 located in the active area of LstiGOBP1 are involved in binding its ligands, probably by forming hydrogen bonds with the ligands [17]. In *Agrilus mali*, hydrogen bond is the main linkage in the binding interaction between AmalOBP8 and geranyl formate, in which Trp106 in AmalOBP8′s binding pocket plays a critical role [18].

In addition to OBPs’ ligand-binding mechanism, there are many studies regarding the ligand-releasing mechanism of OBPs. OBPs are hypothesized to use several mechanisms to release ligands and activate odorant receptors (ORs). Many moth OBPs, such as BmorPBP [19], *Antheraea polyphemus* PBP (ApolPBP) [20], and *Amyelois transitella* PBP (AtraPBP) [21] eject ligands viaa pH-dependent conformational change. The extended C-terminus of those proteins at high or neutral pH can form an α-helix at low pH, which can occupy the binding pocket and push ligands out of the pocket. Some mosquito OBPs, such as *Anopheles gambiae* (AgamOBP1), *Aedes aegypti* (AaegOBP1), and *C. quinquefasciatus* (CquiOBP1), are shorter than moth OBPs, differing mainly in the length of their C-terminus and thus mosquitoes OBPs’ C-terminus cannot form an α-helix to occupy the binding pocket at low pH, but can cover the binding pocket as a lid, which can be opened by disrupting hydrogen bonds at low pH [22,23,24]. Taken together, it can be seen that different OBPs may display distinct interaction mechanisms with theirodor ligands.

*Bactrocera minax* (Enderlein) (Diptera: Tephritidae) is an economically important oligophagous pest damaging citrus fruits and is widely distributed in China. Recently, we reported the functional characterization of two antenna-enriched odorant binding proteins (BminOBP3 and BminOBP6) from *B. minax* [25]. Competitive fluorescence binding experiments demonstrated that both of them showed a remarkable selectivity to the 13 odorants tested. BminOBP3 exhibited strong binding affinity only with undecanol, which is believed to be a major sex pheromone of *B. minax*. However, the interaction mechanism between BminOBP3 and undecanol is not yet clear. In the present study, we combined homology modeling, molecular docking, mutagenesis studies, and binding experiments to explore the mode of action, as well as the binding specificity of BminOBP3. The results of our study revealed the details of the ligand-binding mechanism of BminOBP3 and may provide important insights into the ligand-binding mechanism for inset OBPs.

## 2. Materials and Methods

### 2.1. Chemicals

N-Phenyl-1-naphthylamine(1-NPN)(purity > 98.0%)and1-undecanol (purity > 99.0%) were purchased from TCI corporation, Tokyo, Japan.

### 2.2. 3D Modeling and Molecular Docking

With the amino acid sequence of BminOBP3 as a probe, a blast search was conducted against the current PDB (http://www.rcsb.org accessed on 30 October 2022) to find a template. After searching, the *A. gambiae* odorant binding protein 20 (AgamOBP20) (PDB ID: 3V2L) was chosen as the structural template of BminOBP3. Alignment of BminOBP3 with the template sequence AgamOBP20 was conducted via an online tool (https://www.genome.jp/tools-bin/clustalw accessed on 30 October 2022). The resulting alignment was utilized to build comparative structural modeling [26] by using the SWISS-MODEL server (https://swissmodel.expasy.org/ accessed on 30 October 2022). The modeling rationality was evaluated using the Ramachandran plot [27] and Profile-3D [28]. Based on the built model, the potential binding modes between BminOBP3 and undecanol were analyzed using a CDOCKER [29] docking program. The binding pocket of BminOBP3 was found with reference to the location of the polyethylene glycol (PEG) in AgamOBP20 (PDB ID: 3V2L) by Discovery Studio 2.0. A site sphere radius of 10 Å was set to assign the undecanol binding pocket. The other parameters were set to default. The 3D structure of undecanol was sketched using Discovery Studio 2.0 and further refined using the CHARMm forcefield. After analysis, ten possible binding modes were generated, and the effective binding modes were determined by subsequent mutagenesis studies and fluorescence binding assays.

### 2.3. Cloning and Sequencing of BminOBP3

In our previous study, the BminOBP3 sequence was identified and characterized [30]. To PCR amplify the gene sequence encoding mature BminOBP3, a pair of gene-specific primers, OBP3E-Forward and OBP3E-Reverse (Table 1), were synthesized according to the identified BminOBP3 sequences (Figure S1). The PCR amplification program was as follows: 94 °C for 3 min; 30 cycles of 94 °C for 30 s, 50 °C for 25 s, and 72 °C for 25 s; and a final extension for 5 min at 72 °C. PCR products were examined on a 1% agarose gel, and the target DNA was purified with a Gel Extraction Kit (Promega Bio-Tek, Inc. Madison, USA).The purified products were linked to the Cloning Vector pGEM-T (Promega, Madison, WI, USA) with a 3:1 molar ratio for 12 h at 4 °C and transformed into Trans5α competent cells (TransGen Biotech, Beijing, China) growing on LB solid medium containing ampicillin (50 μg/mL). Positive monoclonal colonies were picked to grow in LB liquid medium with 50 μg/mL ampicillin and confirmed via custom sequencing (BGI, Shenzhen, China).

### 2.4. Preparation of Mutant cDNA Sequences

In this study, we need to construct three mutants of BminOBP3: TOBP3, a mutant that lacks seven amino acids (I116-P122) from C-terminus of BminOBP3; 3D2a, a mutant that lacks the last two amino acids (F121 and P122) from C-terminus of BminOBP3; and V120A, BminOBP3-V120A (valine to alanine at position 120) mutant. The three mutant cDNA sequences were constructed by PCR using the specific primers listed in Table 1 and with the pGEM-T/BminOBP3 vector as a template. For T-OBP3 and 3D2a, the PCR amplification conditions were 94 °C for 3 min; 30 cycles of 94 °C for 30 s, 60 °C/54 °C for 25 s and 72 °C for 25 s; and a final extension for 5 min at 72 °C. PCR products were linked to the Cloning Vector pGEM-T and sequenced by the same protocol mentioned above. The mutant V120A cDNA was generated using theTaKaRa MutanBEST Kit (TaKaRa, Dalian, China)according to the recommended protocol and confirmed by sequencing (BGI). 

### 2.5. Expression Vectors Construction

Sequence-confirmed pGEM-T/*BminOBP3*, pGEM-T/*T-OBP3*, pGEM-T/*3D2a*, pGEM-T/*V120A* and the expression vector pET-32a (+) (Novagen, Darmstadt, Germany) were digested by *Bam*H I and *Hind* III (TaKaRa, Dalian, China) for 1.5 h at 37 °C and then they were separated on an agarose gel, respectively. The target fragments of pGEM-T/*BminOBP3*, pGEM-T/*T-OBP3*, pGEM-T/*3D2a,* and pGEM-T/*V120A* were purified and ligated with pET-32a (+) vector digested with same restriction enzymes, respectively. Recombinant plasmids pET-32a/*BminOBP3*, pET-32a/*T-OBP3*, pET-32a/*3D2a*, and pET-32a/*V120A* were transformed into Trans5α competent cells (TransGen) and cultured on LB solid medium containing ampicillin (50 μg/mL), respectively. Pick positive clones to grow in LB liquid medium with 50 μg/mL ampicillin and confirm it by sequencing (BGI).

### 2.6. Expression and Purification of BminOBP3 and Mutants

Sequence-confirmed pET-32a/*BminOBP3*, pET-32a/*T-OBP3*, pET-32a/*3D2a*, and pET-32a/*V120A* plasmids were extracted using the Plasmid Mini Kit I (Promega) and transformed into BL21 (DE3)-competent cells (TransGen), respectively. The positive clone confirmed by sequencing was added in 1 mL fresh LB liquid medium with 50 μg/mL ampicillin to grow overnight in a shaker set at 37 °C and 200 r/min. The seed culture was added in fresh LB/ ampicillin (50 μg/mL) medium at a ratio of 1:100 to continue growing in the shaker until the OD_600_ value of the bacterial solution reached 0.6–0.8 when isopropyl *β*-D-1-thiogalactopyranoside (IPTG) was used to induce cells to express the recombinant target proteins containing N-terminal His-tags at a concentration of 0.5 mM and continuously incubated for 6 h at 28 °C for BminOBP3, T-OBP3, and V120A and at 37 °C for 3D2a. After a 6 h induction, the bacterial solutions were centrifuged at 4 °C for 10 min at 9820× *g*. Cell pellets werere-suspended in lysis buffer (50 mM Tris-HCI, pH 8.0, 50 mM NaCl, 0.5% TritonX-100, and 2 mg/mL Lysozyme) and then sonicated with an ice-water mixture surrounded. After centrifugation at 17,420×*g* for 15 min at 4 °C, the supernatant and pellet of proteins were collected separately, and sodium dodecyl sulfate-polyacrylamide gel electrophoresis (SDS-PAGE) was used to confirm which part the target protein presented. The supernatant was directly purified by Ni-NTA His•Bind Resin affinity column (7sea PharmatechCo., Shanghai, China) after filtration with 0.22 μm filters (Merck KGaA, Darmstadt, Germany) following the manufacturer’s instructions. The pellet dissolved in solution after denaturation and renaturation with the methods based on the previous study [31]. Recombinant enterokinase (Novoprotein, Shanghai, China) was used to excise the His-tag to avoid its effects on the target protein activity. The concentrations of each protein were measured with a BCA Protein Assay Kit (Solarbio, Beijing, China).

### 2.7. Fluorescence Binding Assays

In order to explore the binding affinities of BminOBP3 and its mutants to undecanol, the binding assays were conducted on a fluorescence spectrophotometer (F-7000; Hitachi, Japan) with parameters set same as the previous study [32]. The fluorescent probe 1-NPN and undecanol were diluted in the HPLC methanol to 1 mM, whereas the protein samples were dissolved in 20 mM Tris-HCL pH 7.4 to obtain 2 μM protein solutions.

To measure the dissociation constant (K_d_) of 1-NPN to BminOBP3 and mutants, aliquots of 1 mM 1-NPN were added to the 2 μM protein solution with a final concentration of 1-NPN ranging from 0 to 22 μM, and the resulting fluorescence intensity values of the maximum fluorescence emission were recorded for the construction of binding curves and Scatchard plots. Bound 1-NPN was determined by fluorescence intensities with the assumption that the protein was 100% active, and it binds the ligands at a 1:1 stoichiometric ratio when saturated. Competitive binding assays were used to judge the affinities of undecanol to each protein. aliquots of 2 μM undecanol were added to the solution containing 2 μM 1-NPN and 2 μM protein with a final concentration of undecanol ranging from 0 to 36 μM. The maximal fluorescence intensity values were plotted against undecanol concentrations. The inhibition constant (K_i_) of each protein was calculated based on the following equation: K_i_ = [IC_50_]/(1 + [1-NPN]/K_1-NPN_). IC_50_ is the concentration of ligand halving initial fluorescence intensity; [1-NPN] is the free concentration of 1-NPN; and K_1-NPN_ is the dissociation constant of the protein/1-NPN complex. All data were collected from three independent measurements.

### 2.8. Statistics Analysis

The significant difference between K_i_ of BminOBP3 with undecanol and K_i_ of T-OBP3 with undecanol was analyzed by Student’s *t*-test using SPSS statistics software 17.0 (SPSS Inc., Chicago, IL, USA).

## 3. Results

### 3.1. 3D Model of BminOBP3

**Figure 1:**
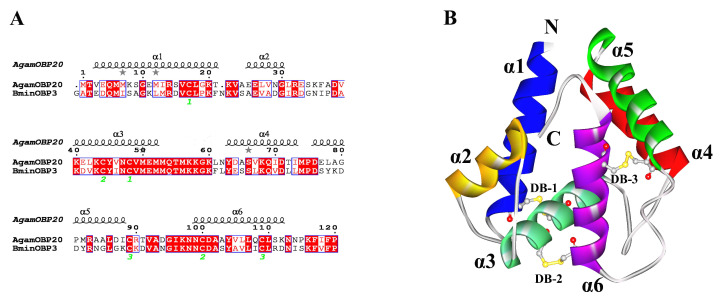
The 3D model of BminOBP3. (**A**) Sequence alignment between AgamOBP20 and BminOBP3. α-helices are displayed as squiggles. The same amino acids between AgamOBP20 and BminOBP3 are highlighted with a red background. Residues with similar physic-chemical properties are displayed in red letters. Three disulfide bridges are labeled with 1–3 below the sequences. (**B**) 3D structure of BminOBP3. The α-helices (α1-6) are color-coded. Three disulfide bridges (DB1-3) are shown as ball and stick models. C is the C-terminus; N is the N-terminus.

With homology modeling to predict protein 3D models, the target sequence and template sequence should have more than 30% sequence identity [26,33,34]. In the present study, we used AgamOBP20 (PDB ID: 3V2L) as the template in the homology modeling of BminOBP3. As shown in Figure 1A, the sequence identity between AgamOBP20 and BminOBP3 is 51.64%. The quality of the homology model was assessed by Ramachandran plot and Profile-3D [27,28]. The result of the Ramachandran plot (Figure S2) demonstrated that 98.36% of all residues of BminOBP3 are in the favored regions, which are greater than the criterion to assess 3D model accuracy (90%), 100% of the residues located in the allowed regions, none of the residues are in disallowed regions. Profile-3D is a model evaluation program based on the “threading” method developed by Professor David Eisenberg at UCLA [28]. The program can assess compatibility between a 3D model and its amino acid sequence. A reasonable model requires that at least 80% of amino acid residues have scores ≥ 0.2 in the Profile-3D. The results (Figure S3) showed that 89.3% of BminOBP3 residues scored above 0.2. The verified score of the 3D model of BminOBP3 is 51.62, which is quite close to the verified expected high score of 53.75. All of these parameters indicate that the predicted model was reliable for BminOBP3. Additionally, the parameters of BminOBP3 are very close to those parameters of its template protein model (AgamOBP20). The result of the Ramachandran plot demonstrated that 99.17% of all residues of AgamOBP20 are in the favored regions. The result of Profile-3D indicated that 97.5% of AgamOBP20 residues score above 0.2.

The predicted 3D model of BminOBP3 consisted of six α-helices and several loops (Figure 1A,B). The six α-helices locate between residues E4-K21 (α1), A27-D35 (α2), K42-M54 (α3), Y65-L75 (α4), D78-K90 (α5) and N101-N115 (α6), in which three disulphide bridges connected C18 in α1and C50 in α3, C46 in α3 and C102 in α6, and C91 in the loop between α5 and α6 and C111 in α6. Five anti-parallel α-helices (α1-α2, α4-α6) converge to form the binding pocket whose wider end is capped by α3, and the pocket’s opposite end is a narrow end, which is open for the ligand entry.

### 3.2. Molecular Docking

**Figure 2:**
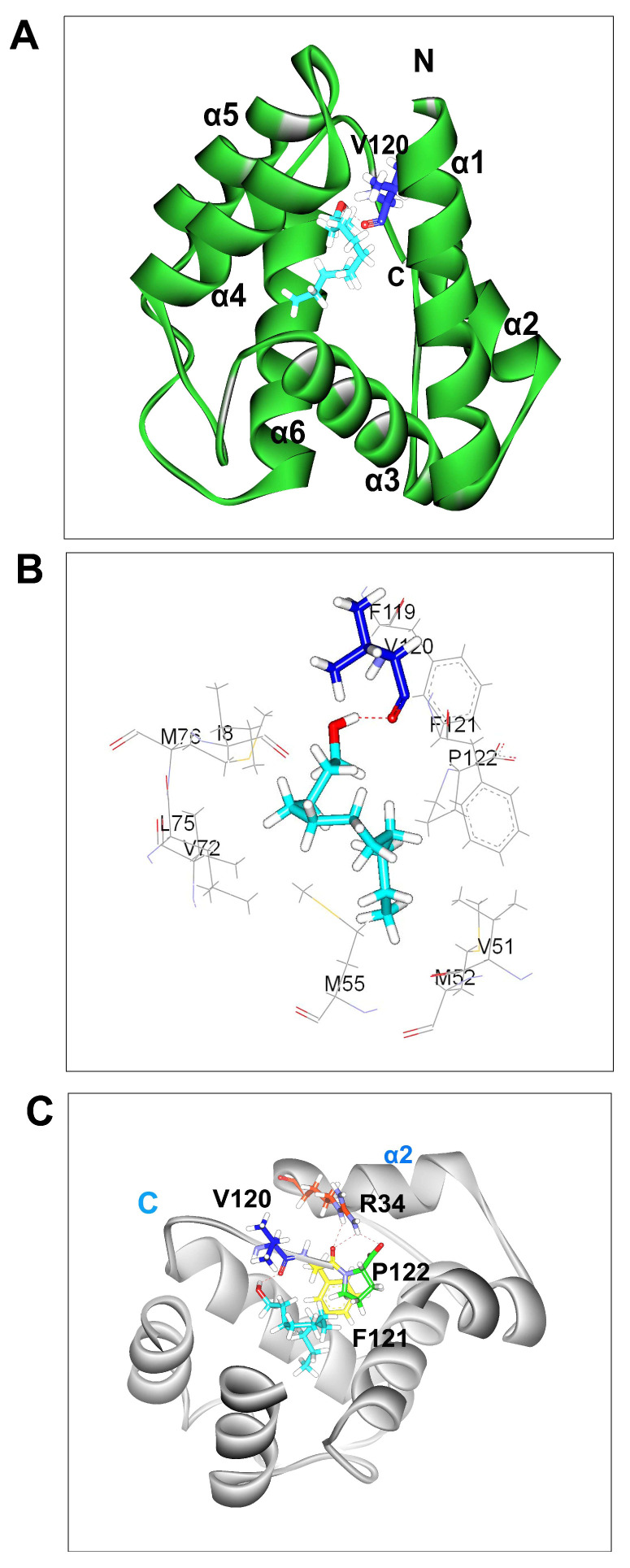
Molecular docking of BminOBP3 to undecanol (conformation 3). (**A**) Binding mode of BminOBP3 with undecanol. Undecanol is displayed as a cyan stick model with hydroxyl oxygen in red. V120 in the C terminal region is shown as a blue stick, which H-bonds to undecanol. The H-bond is shown as a red dotted line. (**B**) Diagram of the hydrophobic interactions of undecanol with key binding site residues. Residues presented as line drawings have a distance to undecanol of less than 4 Å. V120 is displayed as a blue stick. (**C**) Hydrogen bonds in BminOBP3 active area. In the binding mode of BminOBP3 with undecanol, the C-terminus of BminOBP3 not only forms an intermolecular hydrogen bond between V120 and undecanol but also forms intramolecular hydrogen bonds. The R34 in helix-2 separately forms an intramolecular hydrogen bond with F121 and P122 in the C-terminus of BminOBP3.

Our previous study showed that an antenna-enriched odorant binding protein 3 (BminOBP3) was only strongly bound to undecanol among 13 ligands tested [25] (the information regarding 13 ligands see Supplemental Data Table S4). In order to explore the binding reaction between them, undecanol was docked into the pocket of BminOBP3. CDOCKER molecular docking produced ten conformations for BminOBP3/undecanol complex. Three conformations (conformation 1, 2, and 3) of the complex were chosen based on the binding mode. Conformation 1 and 2 are shown in Figures S4A and S5A, respectively. In conformation1 (Figure S4B), the oxygen atom from the hydroxyl group of undecanol forms a hydrogen bond with the hydrogen atom of the hydroxyl group from the side chain of T57 to stabilize undecanol in the binding pocket. In conformation 2 (Figure S5B), the oxygen atom and hydrogen atom from the hydroxyl group of undecanol form hydrogen bonds separately with the hydrogen atom and oxygen atom of the hydroxyl group from the side chain of Y84 to keep undecanol within the binding pocket of BminOBP3. Thus, we deduced that T57 and Y84 are the significant binding sites of BminOBP3. We replaced T57 and Y84 with alanine to generate two mutants T57A and Y84A by using site-directed mutagenesis. After mutagenesis, the mutants were expressed in *Escherichia coli* (Figure S6). The fluorescence binding assays exhibited that both the expressed mutant proteins displayed an obvious increase rather than decrease in affinities to undecanol compared with the wild-type protein (Figure S7 and Table S2), indicating that Conformation 1 and 2 are not an effective conformation. Conformation 3 was an effective conformation, which is shown in Figure 2A, as it was validated by subsequent mutagenesis studies and fluorescence binding assays. In the BminOBP3/undecanol complex, the hydrogen bond is the main linkage between undecanol and BminOBP3. The hydrogen atom from the hydroxyl group of undecanol forms a hydrogen bond with the oxygen atom of the carbonyl group from the main chain of V120 in the C-terminal region of BminOBP3 (Figure 2C). Meanwhile, the F121 and P122 in the C-terminus of BminOBP3 form three intramolecular hydrogen bonds with the R34 in helix-2, which acts like a ‘lock’, which locks down BminOBP3’s C-terminus and restrains undecanol in the inner binding pocket of BminOBP3 (Figure 2C). These hydrogen bonds occur between the carbonyl oxygen of F121 and carboxyl oxygen of P122 in the backbone as hydrogen bond acceptors and the (-NH-) group from the side chain of R34 as hydrogen bond donors (Figure 2C).

In addition to hydrogen bonds, hydrophobic interactions may also play a significant role in the binding interaction between BminOBP3 and undecanol. The hydrophobic residues within 4 Å to undecanol are shown in Figure 2B. In the complex, those residues include I8, V51, M52, M55, V72, L75, M76, F119, V120, F121, and P122 (Figure 2B).

### 3.3. Expression and Purification of BminOBP3 and Mutant Proteins

**Figure 3:**
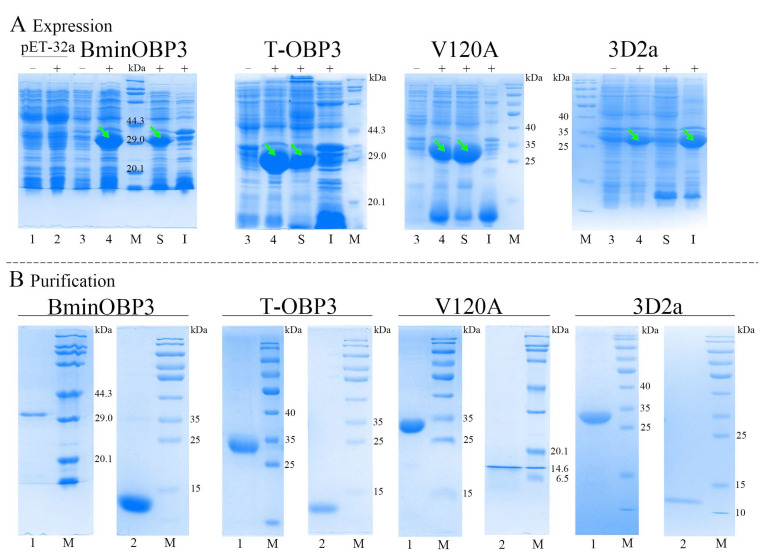
Expression and purification of BminOBP3 and its mutants, analyzed by SDS-PAGE. (**A**) Recombinant expression of BminOBP3 and its three mutants TOBP3, V120A, and 3D2a in *E. coli* BL21 (ED3). Lane 1 and 2: The crude expression production of pET32a vectors that are not inserted target genes, which was used as control; Lane 3 and 4: the crude expression production of recombinant vectors pET32a/BminOBP3, pET32a/T-OBP3, pET32a/V120A and pET32a/3D2a; S: The supernatant of the crude expression production of the recombinant vectors; I: Inclusion body of the crude expression production of the recombinant vectors; M: Protein molecular mass marker; − and +: *E. coli* cells before and after IPTG induction; The green arrows indicate the target bands.. BminOBP3: the wild-type protein; TOBP3: A mutant that lacks seven amino acids (I116-P122) from the C-terminus of BminOBP3; V120A: BminOBP3-Val120A (valine to alanine at position 120) mutant; 3D2a: A mutant that lacks the last two amino acids (F121 and P122) from the C-terminus of BminOBP3; (**B**) Purification of recombinant BminOBP3 and its mutants TOBP3, V120A and 3D2a. 1: Ni-NTA affinity-purified recombinant BminOBP3 and mutants; 2: Re-purification of BminOBP3 and mutants after His-tag removal via recombinant enterokinase.

Molecular docking results suggested that the C-terminal segment (I116-P122), especially the last three amino acids of the C-terminus (V120-P122), play an essential role in the ligand binding for BminOBP3. To confirm their function, BminOBP3 (the wild-type protein) and three mutants T-OBP3, 3D2a, and V120A (for the meaning of those mutants, see Table 2) were built. The wild-type protein sequence of BminOBP3 is reported in Figure S1. As shown in Figure 3A, all recombinant proteins including BminOBP3 and mutants were successfully expressed in *E. coli* cells after the induction of IPTG, and the specific bands of the expected size (~32 kDa) corresponding to each recombinant protein were detected by SDS-PAGE (Figure 3A, lane 4), yet these bands did not appear or were very weak when bacterial cells were not induced by IPTG (Figure 3A, lane 3). Meanwhile, an expression vector pET-32a that does not have target genes was used as control without (Figure 3A, lane 1) and with (Figure 3A, lane 2) induction of IPTG. Further analysis demonstrated that the recombinant BminOBP3, T-OBP3, and V120A were mainly expressed in the supernatants (Figure 3A, lane S). In contrast, 3D2a was expressed mainly in inclusion bodies (Figure 3A, lane I), whose denaturation and renaturation were carried out based on the methods of the previous study [30]. Subsequently, Ni-NTA His•bind Resin columns were used to obtain the purified recombinant proteins of BminOBP3 and mutants (Figure 3B, lane1). The yields of the purified proteins were 0.80, 0.31, 0.52, and 1.06 mg/mL for BminOBP3, T-OBP3, 3D2a, and V120A, respectively. To avoid the possible influences of His-tags on subsequent research, the tags were excised via recombinant enterokinase. After digestion, the target proteins whose His-tags were removed were checked by SDS-PAGE (Figure 3B, lane 2), upon which the expected bands of 13–14 kDa were found, which was consistent with the theoretical molecular weight of 13.62 kDa for BminOBP3, 12.8kDa for T-OBP3, 13.89 kDa for V120A, and 13.37 kDa for 3D2a.

### 3.4. Binding Affinities of BminOBP3 and Mutants to Undecanol

**Figure 4:**
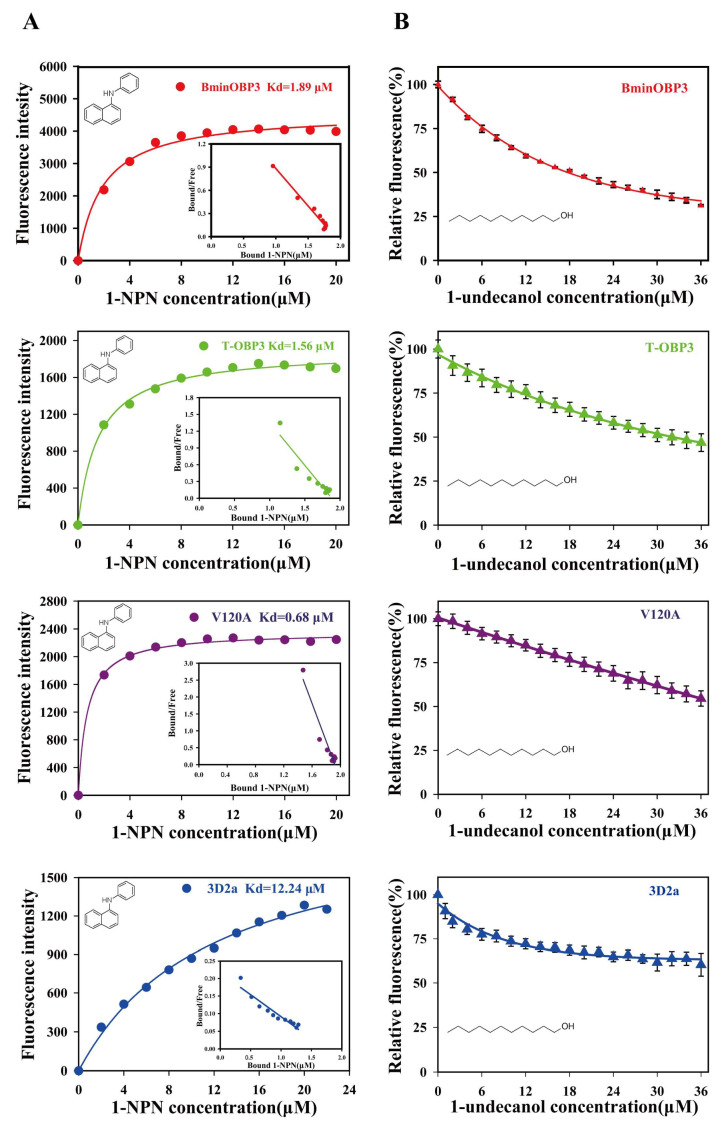
Ligand binding assays of BminOBP3 and its mutants TOBP3, V120A, and 3D2a. (**A**) Binding curves of 1-NPN to BminOBP3 and its mutants and relative Scatchard plots (inset). (**B**) Fluorescence completive binding curves of BminOBP3 and mutants to undecanol. Data points are shown as mean ± SEM (N = 3).

To determine the binding affinities of BminOBP3 and mutants to undecanol, we first measured the dissociation constant (K_d_) between proteins and 1-NPN fluorescence reporter. The results showed that all proteins tested could bind 1-NPN and emit strong fluorescence. Based on the maximal fluorescence intensity changes (Figure 4A), K_d_ values of each protein/1-NPN complex were calculated as follows: 1.89 μM for BminOBP3/1-NPN, 0.68 μM for V120A/1-NPN, 1.56 μM for T-OBP3/1-NPN, and 12.24 μM for 3D2a/1-NPN (Figure 4A).

Next, we investigated the ability of undecanol to displace 1-NPN from BminOBP3/, T-OBP3/, 3D2a/, or V120A/1-NPN complex by using the competitive binding assay, respectively (Figure 4B). The values of IC_50_ and K_i_ for all proteins to undecanol were reported in Table 2. As shown in Table 2 and Figure 4B, the mutant T-OBP3 exhibited a significant decrease in its affinity for undecanol compared with BminOBP3, indicating that the C-terminus is of importance for BminOBP3 to bind undecanol. The mutants V120A and 3D2a completely lost their binding capacity to undecanol. Undecanol cannot replace the 50% 1-NPN from V120A/ and 3D2a/1-NPN complex even when its concentration reaches 36 μM (Figure 4B), which suggests that V120, F121, and P122 play a crucial role in the interaction between BminOBP3 and undecanol.

## 4. Discussion

In our previous studies, we had shown that *B. minax* OBP3 (*BminOBP3*) was highly expressed in male and female antennae [30], and the results of fluorescent competitive binding assays demonstrated that BminOBP3 exhibited strong binding affinity only with undecanol among 13 ligands tested [25]. Undecanol is believed to be a main *B. minax* pheromone [35]. Those results indicated that BminOBP3 plays a significant role in the olfactory recognition of *B. minax*. However, those studies are not enough to understand the ligand-binding mechanisms of BminOBP3.

In the present study, a 3D model of BminOBP3 was generated with homology modeling based on the structure of AgamOBP20. Undecanol was chosen to dock with the BminOBP3 3D model. The dock experiments indicated that BminOBP3’s binding pocket is formed mainly by hydrophobic amino acids including I8, V51, M52, M55, V72, L75, M76, F119, V120, F121, and P122 (Figure 2B). Accumulated studies suggest that hydrogen bonds are the key force in OBPs binding to their ligands [15,16,17,18]. In the BminOBP3/ undecanol complex, four significant hydrogen bonds were detected (Figure 2C). One is an intermolecular hydrogen bond between BminOBP3 and undecanol. V120 in the C-terminal region of BminOBP3 makes a specific hydrogen bond with undecanol. The other three are intramolecular hydrogen bonds. F121 and P122 in the C-terminus of BminOBP3 form three hydrogen bonds with the side chains of R34 in helix-2. We infer that the three amino acids (V120, F121, and P122) in the C-terminus of BminOBP3 are quite significant sites for ligand binding of BminOBP3.

To confirm the hypothesis mentioned above, mutants of BminOBP3 were used to explore the ligand-binding mechanism of BminOBP3. The results of fluorescent competitive binding assays showed that mutant V120A (valine to alanine at position 120) completely lost its binding affinity (Table 2 and Figure 4B), thus implying that V120 in the C-terminus of BminOBP3 is the critical residue for BminOBP3 binding with undecanol. It is well known that many insect OBPs’ C-terminus is a random coil, such as *Apis mellifera* pheromone-binding protein (AmelPBP) (PDB ID: 3BFH), AgamOBP20 (PDB ID: 3V2L), AaegOBP1 (PDB ID: 3K1E), and so on, which is a flexible secondary protein structure, at the opening of BminOBP3 binding pocket. In this regard, the V120 in the C-terminus of BminOBP3 is quite easy to first collide with odors within sensillar lymph compared with those residues situated inside the binding cavity, which indicates that V120 is most likely to contribute to the initial ligand recognition. In fact, numerous studies have demonstrated that those amino acids that are located at the entrance of the OBP binding pocket participate in the initial ligand recognition and determine OBPs’ ligand-binding specificity. For example, T57 of LUSH [16], T15 and W42 of LstiGOBP1 [17], and N74 of LmigOBP1 [36], all of which are situated at the entrance of the OBP binding pocket and interact with its specific ligands via hydrogen bonds, determine these OBPs’ ligand-binding specificity. Furthermore, our result may provide important insight into the ligand uploading process of BminOBP3. The current studies on insect OBPs mainly focus on their static properties, such as 3D structures and OBPs’ ligand-binding affinities, the dynamic interaction between insect OBP and its ligands remains largely unclear. To our knowledge, Gong et al. are the first research group that conducted studies on the dynamic interaction between insect OBP and its ligands [37,38], which suggested that the ligand uploading process of OBPs was a stepwise binding process: (1) the ligand is first associate with the OBP on an external site, and then (2) slow embedded in the internal pocket. The authors propose that the external site is a hydrophobic patch on the surface of OBP, specifically a site near OBP’s C-terminus. However, the exact position of the external site was unknown. In BminOBP3, the V120 in the C-terminus of BminOBP3 may be the external binding site, on which the undecanol can be initially bound by a hydrogen bond. Upon undecanol binding, undecanol is embedded into the internal pocket of BminOBP3. In the process, the C-terminus plays an important role because the C-terminus truncated BminOBP3 (T-OBP3), which lacks seven residues from the C-terminus, displayed a significant decrease in binding to undecanol (Table 2 and Figure 4B). A possible explanation is that the C-terminus of BminOBP3 can guide undecanol to the inner binding pocket and can properly orientate it in the inner binding pocket. When undecanol enters the inner binding pocket of T-OBP3, the efficient binding between T-OBP3 and undecanol will be limited due to lacking navigation of the C-terminus, although the C-terminus removal may lead to a lower barrier for undecanol to enter the inner binding pocket of T-OBP3.

The last two amino acids (F121 and P122) in the C-terminus of BminOBP3 form three intramolecular hydrogen bonds with the side chains of R34 in helix-2. Comparing our results with the similar results of previous studies on other Diptera OBPs, such as AgamOBP1 [22], AaegOBP1 [24], and CquiOBP1 [23], it can be concluded that the intramolecular hydrogen bonds in OBPs’ C-terminus are also critical for ligand binding of OBPs. In the mutant 3D2a, which lacks the last two amino acids (F121 and P122) from the C-terminus of BminOBP3, the binding to undecanol is also completely abolished, implying that F121 and P122 are also crucial for BminOBP3′s ligand binding. Even so, we suggest that the role of F121 and P122 in ligand binding of BminOBP3 is different from V120. F121 and P122 may act as a ‘lock’, which locks down BminOBP3′s C-terminus and restrains undecanol in the inner binding pocket by hydrogen bonds to the side chains of R34 in helix-2 (Figure 2C). This ‘lock’ also can be observed in the other Dipteran OBP. For instance, in CquiOBP1, the intramolecular hydrogen bonds between H23, Y54, and the C-terminal residue V125 locks the C-terminus of CquiOBP1 and restrains the oviposition pheromone (*5R*,*6S*)-6-acetoxy-5-hexadecanolide (MOP) in the inner binding cavity [23]. The authors suggested that these intramolecular hydrogen bonds are acid-labile, which can be disrupted at low pH, causing the C-terminal loop to open, thereby lowering binding affinity for ligand, which is even believed to be a common ligand release mechanism [22,23]. To confirm this, we also investigate the pH effect on the binding affinity of BminOBP3 with undecanol in this study. Our results demonstrated that the affinity between BminOBP3 and undecanol was not completely abolished, although the affinity exhibited a significant decrease under acidic pH 5.0 compared with that under pH 7.4 (Figure S8 and Table S3). One potential explanation is that BminOBP3 may act as a scavenger, just as previous studies suggested [2,39,40,41]. Those studies suggested that some OBPs can act as a scavenger to provide a molecular form of gain control or to inactivate odorant signals by sequestering odorants from the sensillar lymph and the receptors. Based on those studies, it seems that those OBPs would not be needed for releasing its ligands. Undoubtedly, further investigation is needed to explore the ligand-releasing mechanisms of BminOBP3.

## 5. Conclusions

Based on the results of this study, we suggest that the C-terminus (I116-P122) of BminOBP3, especially the last three amino acids of the C-terminus (V120-P122), play an essential role in the ligand-binding for BminOBP3. The information from this research not only promotes our understanding of the interaction mechanism between BiminOBP3 and undecanol but also can facilitate the discovery and design of novel active semiochemicals.

## Figures and Tables

**Table 1:** Primers used for expressing BminOBP3 and its mutants T-OBP3, 3D2a, and BminOBP3-V120A.

Purpose		Primer Name	Primer Sequence (5′-3′)
Cloning/expression for the wild-type BminOBP3		OBP3E-Forward	CGC**GGATCC**G_(1)_GTGCTACGGAAGATC_(16)_
		OBP3E-Reverse	CCC**AAGCTT**T_(369)_TAAGGGAAAACAAAC_(354)_
Expression for mutants	For the expression of the C-terminus (I116-P122) truncated mutant of BminOBP3	T-OBP3E-Forward	CGC**GGATCC**G_(1)_GTGCTACGGAAGA_(14)_
		T-OBP3E-Reverse	CCC**AAGCTT**TTAA_(354)_TTATCGCGCAAACA_(331)_
	For the expression of the mutant that lacks the last two amino acids (F121 and P122) from the C-terminus of BminOBP3	3D2aE-Forward	**GGATCC**G_(1)_GTGCTACGGAA_(12)_
		3D2aE-Reverse	**AAGCTT**TTAA_(360)_ACAAACTTGGATATATT_(343)_
	For the expression of the BminOBP3-V120A (valine to alanine at position 120) mutant	V120A-Forward	A_(348)_TCCAAGTTTGCTTTCCCTTAA_(369)_
		V120A-Reverse	A_(347)_TATTATCGCGCAAACAAATTAG_(323)_

The bold underlined letters in the primers indicate the restriction site for the enzymes *Bam*H Ⅰ (forward primers) and *Han*d Ⅲ (reverse primers). The numbers marked after the base in each primer indicate the positions of these bases on the nucleotide sequence. The red GCT shows the amino acid substitution: V120(GTT)-A(GCT).

**Table 2:** Binding affinities of BminOBP3 and its mutants to undecanol in fluorescence competitive binding assays.

Proteins	Undecanol	
	IC_50_ (μM)	K_i_ (μM)
BminOBP3	17.72 ± 0.78	11.59 ± 0.51 a
T-OBP3	32.06 ± 0.74	19.57 ± 0.45 b
V120A	>36	_
3D2a	>36	_

K_i_ (inhibition constant) was calculated from the corresponding IC_50_ (the concentration of ligand when 1-NPN was replaced by half); ‘>36’ shows that the IC_50_ values could not be obtained with the tested ligand concentrations; ‘–’ means the K_i_ values could not be calculated. BminOBP3: the wild-type protein; TOBP3: a mutant that lacks seven amino acids (I116-P122) from the C-terminus of BminOBP3; 3D2a: a mutant that lacks the last two amino acids (F121 and P122) from the C-terminus of BminOBP3; V120A: BminOBP3-V120A (valine to alanine at position 120) mutant. K_i_ values within a column followed by a different letter are significantly different [mean ± SE, Student’s *t-*test, *p* < 0.05].

## Data Availability

Data is contained within this article and the supplementary material.

## Supplementary Materials

The following supporting information can be downloaded at www.mdpi.com/xxx/s1,Figure S1. cDNA sequence and deduced amino acid sequence of BminOBP3; Figure S2. Ramachandran plot of the BminOBP3 model; FigureS3. Profile 3D score of the BminOBP3 model; FigureS4. MoleculardockingofBminOBP3toundecanol(Conformation1); FigureS5. MoleculardockingofBminOBP3toundecanol(Conformation2); Figure S6. SDS-PAGE analysis of expression and purification of BminOBP3, T57A and Y84A; Figure S7. Ligand binding assay of BminOBP3 and its mutants T57A and Y84A; Figure S8. Ligand binding assay of BminOBP3 at pH 5.0 and pH 7.4 condition; Table S1. Primers used for expressing BminOBP3 and its mutants BminOBP3-T57A and BminOBP3-Y84A; Table S2. Binding affinities of BminOBP3, T57A, and Y84A to undecanol in fluorescence competitive binding assays; Table S3. Binding affinities of BminOBP3 to 1-undecanol in fluorescence competitive binding assays at pH7.4 and pH5.0; Table S4. The source of 13 ligands used in the study reported by Chen et al., 2021 [25].
