# Supplementary material for: Key Amino Acid Residues Involved in Binding Interactions between Bactrocera minax Odorant-Binding Protein 3 (BminOBP3) and Undecanol
Source: Insects. 2023 Sep 5;14(9):745. doi: 10.3390/insects14090745 (PMC10531759; doi:10.3390/insects14090745)
Supplement: Supplementary file 1 [file insects-14-00745-s001.zip › insects-2521950-original image.pdf]

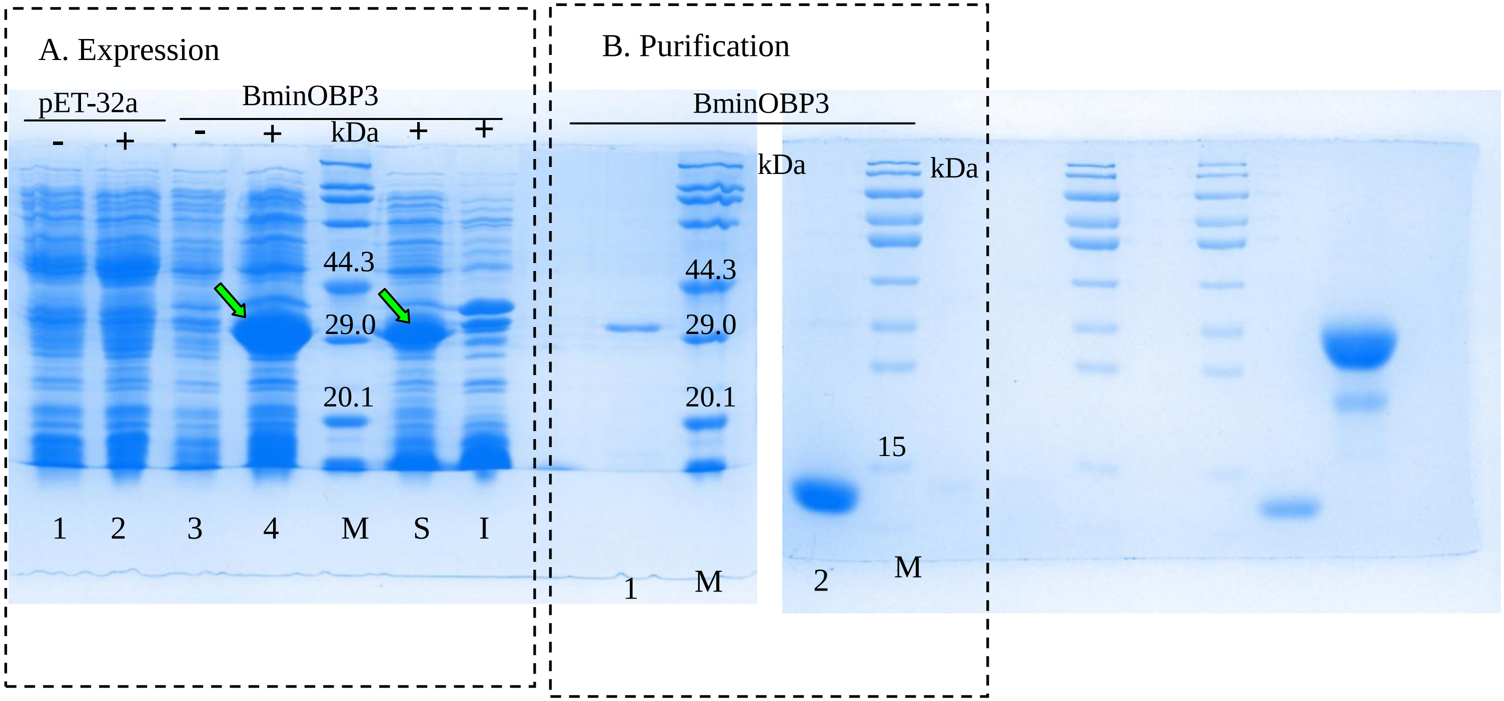

Figure 1. **Expression and purification of BminOBP3** (the wild type protein)**, analyzed by SDS-PAGE. (A) Recombinant expression of BminOBP3 in *Escherichia coli* BL21 (ED3).** Lane 1 and 2: The crude expression production of pET32a vectors that are not inserted target genes, which was used as control; Lane 3 and 4: the crude expression production of recombinant vectors pET32a/BminOBP3; S: The supernatant of the crude expression production of the recombinant vectors; I: Inclusion body of the crude expression production of the recombinant vectors; M: Protein molecular mass marker; – and +: *E. coli* cells before and after IPTG induction; arrow heads indicate the target bands. **(B) Purification of recombinant BminOBP3**. 1: Ni-NTA affinity-purified recombinant BminOBP3; 2: Re-purification of BminOBP3 after His-tag removal via recombinant enterokinase.

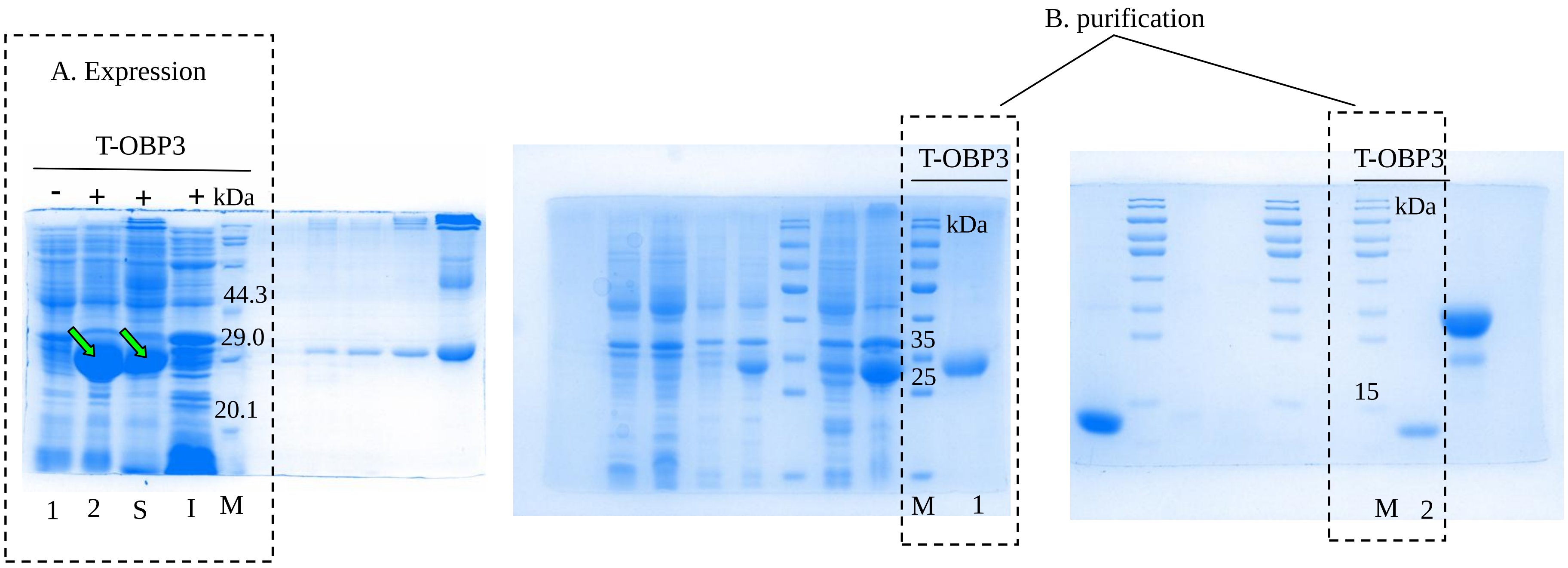

Figure 2. **Expression and purification of TOBP3 (**A mutant that lacks seven amino acids (I116-P122) from the C-terminus of BminOBP3**), analyzed by SDS-PAGE. (A) Recombinant expression of TOBP3 in *Escherichia coli* BL21 (ED3)**. Lane 1 and 2: the crude expression production of recombinant vectors pET32a/TOBP3; S: The supernatant of the crude expression production of the recombinant vectors; I: Inclusion body of the crude expression production of the recombinant vectors; M: Protein molecular mass marker; – and +: *E. coli* cells before and after IPTG induction; arrow heads indicate the target bands. **(B) Purification of recombinant TOBP3**. 1: Ni-NTA affinity-purified recombinant TOBP3; 2: Re-purification of TOBP3 after His-tag removal via recombinant enterokinase.

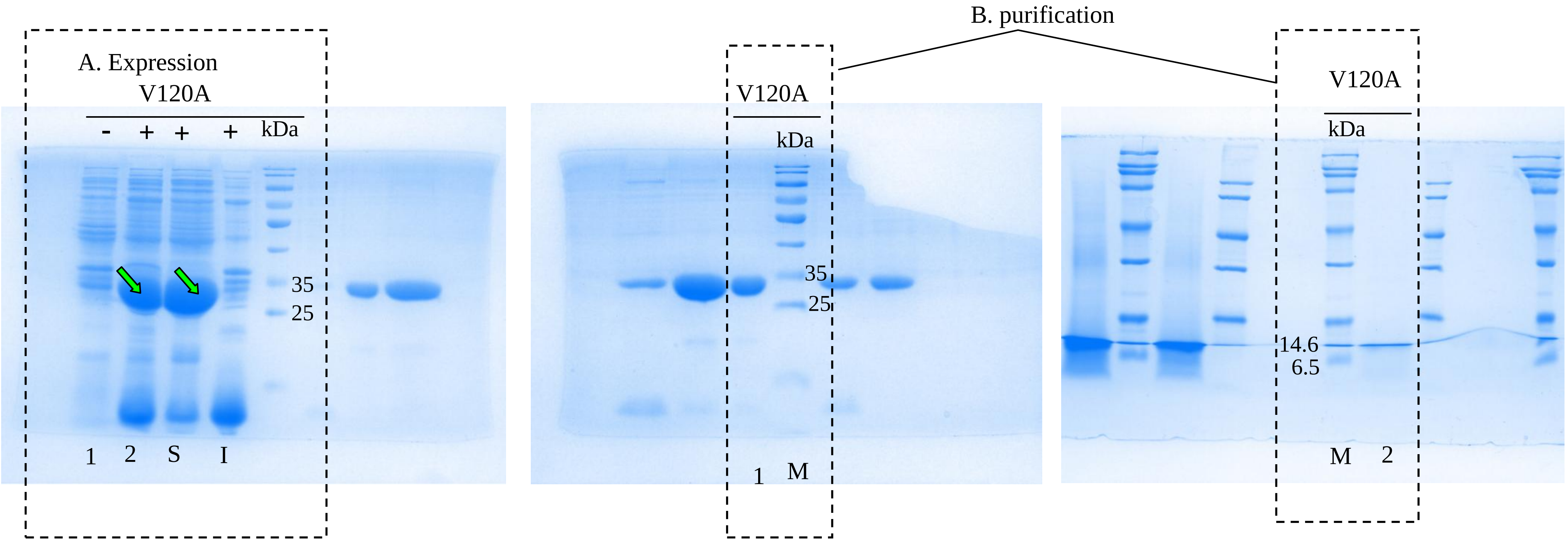

Figure 3. **Expression and purification of V120A (**BminOBP3-Val120A (valine to alanine at position 120) mutant**), analyzed by SDS-PAGE. (A) Recombinant expression of V120A in *Escherichia coli* BL21 (ED3)**. Lane 1 and 2: the crude expression production of recombinant vectors pET32a/ V120A; S: The supernatant of the crude expression production of the recombinant vectors; I: Inclusion body of the crude expression production of the recombinant vectors; M: Protein molecular mass marker; – and +: *E. coli* cells before and after IPTG induction; arrow heads indicate the target bands. **(B) Purification of recombinant V120A**. 1: Ni-NTA affinity-purified recombinant V120A; 2: Re-purification of V120A after His-tag removal via recombinant enterokinase.

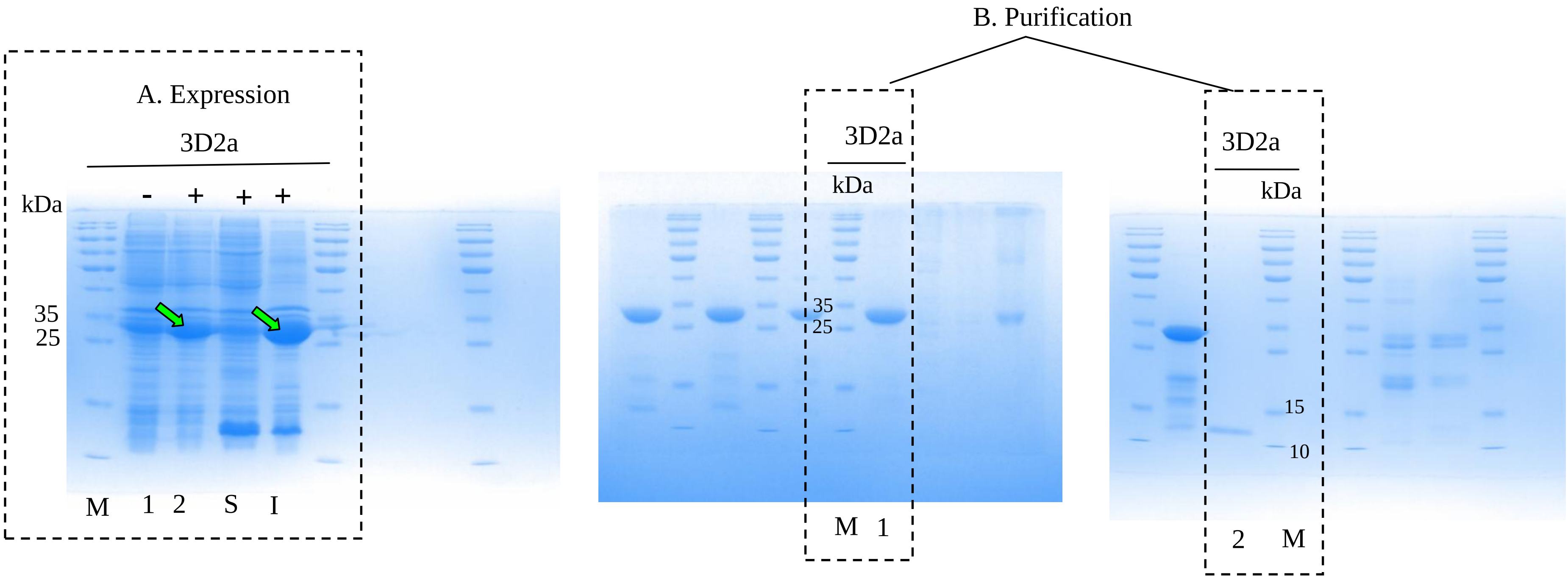

Figure 4. **Expression and purification of 3D2a (**A mutant that lacks the last two amino acids (F121 and P122) from the C-terminus of BminOBP3**), analyzed by SDS-PAGE. (A) Recombinant expression of 3D2a in *Escherichia coli* BL21 (ED3)**. Lane 1 and 2: the crude expression production of recombinant vectors pET32a/3D2a; S: The supernatant of the crude expression production of the recombinant vectors; I: Inclusion body of the crude expression production of the recombinant vectors; M: Protein molecular mass marker; – and +: *E. coli* cells before and after IPTG induction; arrow heads indicate the target bands. **(B) Purification of recombinant 3D2a**. 1: Ni-NTA affinity-purified recombinant 3D2a; 2: Re-purification of 3D2a after His-tag removal via recombinant enterokinase.

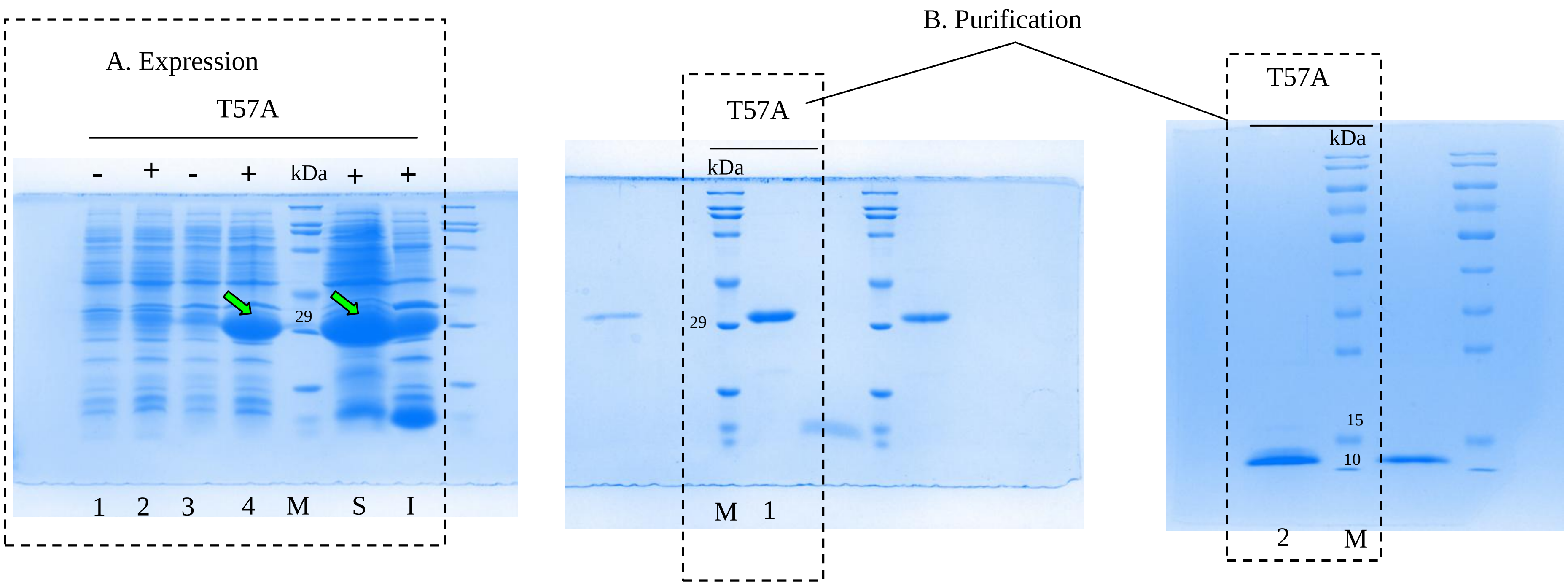

Figure 5. **SDS-PAGE analysis of expression and purification of T57A**（BminOBP3-T57A (threonine to alanine at position 57) mutant）**. (A) Expression of recombinant proteins of T57A expressed in *Escherichia coli* BL21(DE3) cells.** Lane 1 and 2: The crude expression production of pET-32a vectors that not inserted target genes was used as control; Lane 3 and 4: the crude expression production of recombinant vectors pET-32a/ T57A; S: The supernatant of the crude expression production of the recombinant vectors; I: Inclusion body of the crude expression production of the recombinant vectors; M: Protein molecular mass marker; – and +: *E. coli* cells before and after IPTG induction; arrows indicate the target bands. (B) **Purification of recombinant T57A.** Lane 1: Ni-NTA affinity-purified recombinant T57A; Lane 2: Re-purification of T57A after His-tag removal via recombinant enterokinase. M: Protein molecular mass marker.

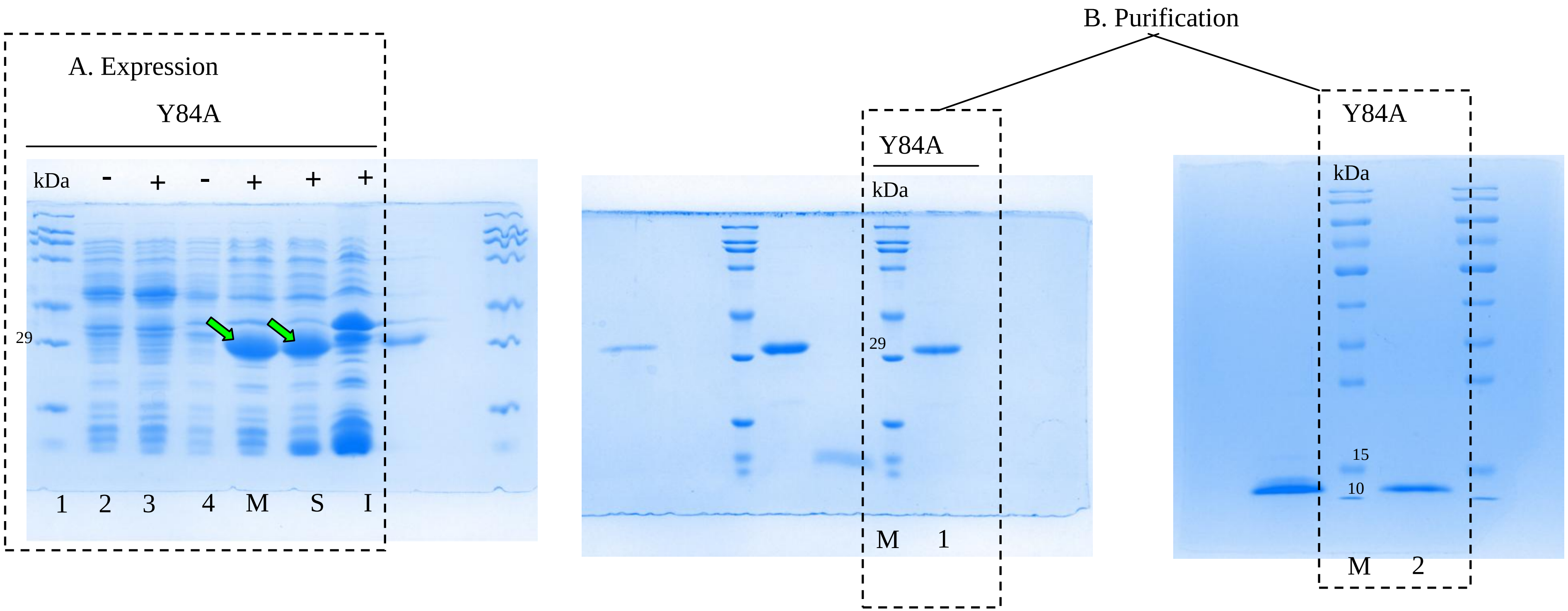

Figure 6. **SDS-PAGE analysis of expression and purification of Y84A**（BminOBP3-Y84A (tyrosine to alanine at position 84) mutant）. **(A) Expression of recombinant proteins of Y84A expressed in *Escherichia coli* BL21(DE3) cells**. Lane 1 and 2: The crude expression production of pET-32a vectors that not inserted target genes was used as control; Lane 3 and 4: the crude expression production of recombinant vectors pET-32a/ Y84A; S: The supernatant of the crude expression production of the recombinant vectors; I: Inclusion body of the crude expression production of the recombinant vectors; M: Protein molecular mass marker; – and +: *E. coli* cells before and after IPTG induction; arrows indicate the target bands. **(B) Purification of recombinant Y84A.** Lane 1: Ni-NTA affinity-purified recombinant Y84A; Lane 2: Re-purification of Y84A after His-tag removal via recombinant enterokinase. M: Protein molecular mass marker.
