# Supplementary material for: Key Amino Acid Residues Involved in Binding Interactions between Bactrocera minax Odorant-Binding Protein 3 (BminOBP3) and Undecanol
Source: Insects. 2023 Sep 5;14(9):745. doi: 10.3390/insects14090745 (PMC10531759; doi:10.3390/insects14090745)
Supplement: Supplementary file 1 [file insects-14-00745-s001.zip › insects-2521950-Supplementary material.pdf]

Figure S1. cDNA sequence and deduced amino acid sequence of BminOBP3. The start and stop codon are boxed. Putative signal peptide at the N-terminus is underlined. The six conserved cysteine residues are circled.

Figure S2. Ramachandran plot of the BminOBP3 model. The regions surrounded by the blue line in the plot are the favored regions. The regions surrounded by the purple line in the plot are the allowed regions except the favored regions. The other regions in the plot except the favored regions and allowed regions are the disallowed regions.

Figure S3. Profile 3D score of the BminOBP3 model. The result showed that 97.5 % of BminOBP3 residues scored above 0.2.

Figure S4. Molecular docking of BminOBP3 to undecanol (Conformation 1). (A) Binding mode of BminOBP3 with undecanol. Undecanol is displayed as a cyan stick model with the hydroxyl oxygen in red. T57 in the loop region between α3 and α4 is shown as blue stick, which H-bonds to undecanol. The H-bond is shown as a red dotted line. (B) The orientation and conformation of undeca -nol and hydrogen bond reaction in BminOBP3 active area.

Figure S5. Molecular docking of BminOBP3 to undecanol (Conformation 2).
(A) Binding mode of BminOBP3 with undecanol. Undecanol is displayed as a cyan stick model with the hydroxyl oxygen in red. Y84A in the α5 is shown as blue stick, which H-bonds to undecanol. The H-bond is shown as red dotted lines. (B) The orientation and conformation of undecanol and hydrogen bond reactions in BminOBP3 active area.

Figure S6. SDS-PAGE analysis of expression and purification of BminOBP3, T57A and Y84A.
(A) Expression of recombinant proteins of BminOBP3, T57A and Y84A expressed in *Escherichia coli* BL21(DE3) cells. Lane 1 and 2: The crude expression production of pET-32a vectors that not inserted target genes was used as control; Lane 3 and 4: the crude expression production of recombinant vectors pET-32a/BminOBP3, pET-32a/T57A and pET-32a/Y84A; S: The supernatant of the crude expression production of the recombinant vectors; I: Inclusion body of the crude expression production of the recombinant vectors; M: Protein molecular mass marker; – and +: *E. coli* cells before and after IPTG induction; arrows indicate the target bands. OBP3: the wild type protein-BminOBP3; T57A: BminOBP3-T57A (threonine to alanine at position 57) mutant; Y84A: BminOBP3-Y84A (tyrosine to alanine at position 84) mutant; (B) Purification of recombinant BminOBP3 and its mutants T57A and Y84A. Lane 1: Ni-NTA affinity-purified recombinant BminOBP3 and mutants; Lane 2: Re-purification of BminOBP3 and mutants after His-tag removal via recombinant enterokinase. M: Protein molecular mass marker.

Figure S7. Ligand binding assay of BminOBP3 and its mutants T57A and Y84A. (A) The binding curves and Scatchard plots (inset) of 1-NPN to BminOBP3 and its mutants. (B) Fluorescence competitive binding curves of BminOBP3 and its mutants to undecanol. Data points are shown as mean ± SEM (*N*=3).

Figure S8. Ligand binding assay of BminOBP3 at pH 5.0 and pH 7.4 condition. (A) The binding curves and Scatchard plots (inset) of 1-NPN to BminOBP3. (B) Fluorescence competitive binding curves of BminOBP3 and its mutants to 1-undecanol. Data points are shown as mean ± SEM (*N*=3). Different background colors indicating different pH environment, in which the ligand binding assays were carried out.

Figure S1

>BminOBP3

```
1      ATGTTGAATAAAATAAATCCTTTCATCCTTACTGCTGTATTCATCGCCTTGGTTTTACATTCGGATCAAGTTTCCGGCGGTGCTACGGAA
1       M  L  N  K  I  N  P  F  I  L  T  A  V  F  I  A  L  V  L  H  S  D  Q  V  S  G  G  A  T  E

91     GATCAAATGATATCTGCTGGAAAACTGATGCGTGATGTGTGTTTGCCGAAATTTAATAAGGTATCAGCAGAAGTGGCAGATGGCATCAGA
31      D  Q  M  I  S  A  G  K  L  M  R  D  V  C  L  P  K  F  N  K  V  S  A  E  V  A  D  G  I  R

181    GATGGTAATATTCCAGACGCGAAGGACGTAAAATGTTATATTAATTGTGTGATGGAAATGATGCAAACAATGAAGAAAGGCAAGTTTCTT
61      D  G  N  I  P  D  A  K  D  V  K  C  Y  I  N  C  V  M  E  M  M  Q  T  M  K  K  G  K  F  L

271    TATGAAAGCTCATTGAAACAAGTTGATTTGCTGATGCCGGACAGTTACAAGGATGATTACCGCAACGGGCTAGGAAAATGCAAAGATGTA
91      Y  E  S  S  L  K  Q  V  D  L  L  M  P  D  S  Y  K  D  D  Y  R  N  G  L  G  K  C  K  D  V

361    GCAAATGGAATAAAGAACAATTGCGACGCCTCCTACGCTGTTCTAATTTGTTTGCGCGATAATATATCCAAGTTTGTTTTCCCTTAA
121     A  N  G  I  K  N  N  C  D  A  S  Y  A  V  L  I  C  L  R  D  N  I  S  K  F  V  F  P  *
```

Figure S2

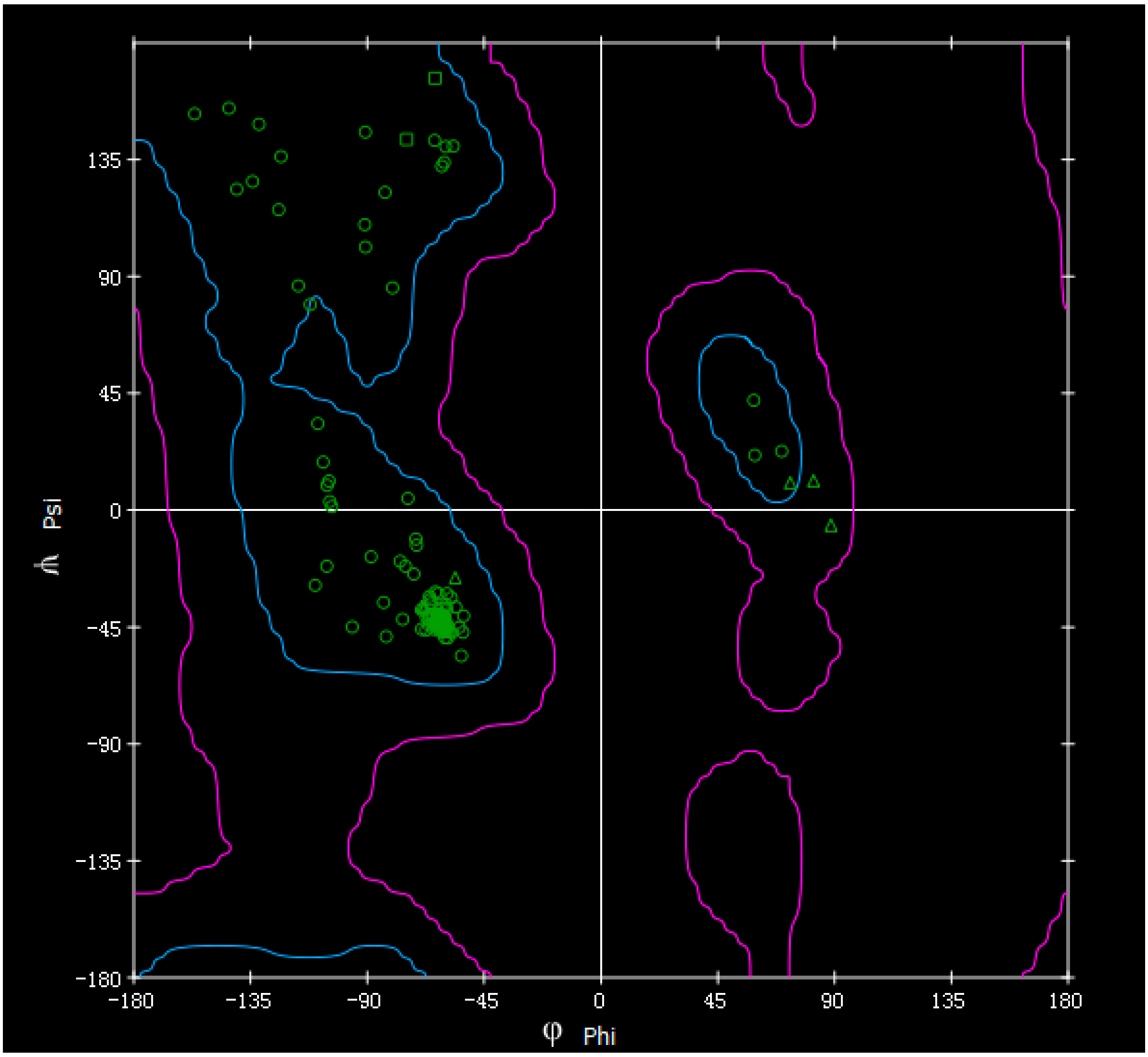

Figure S3

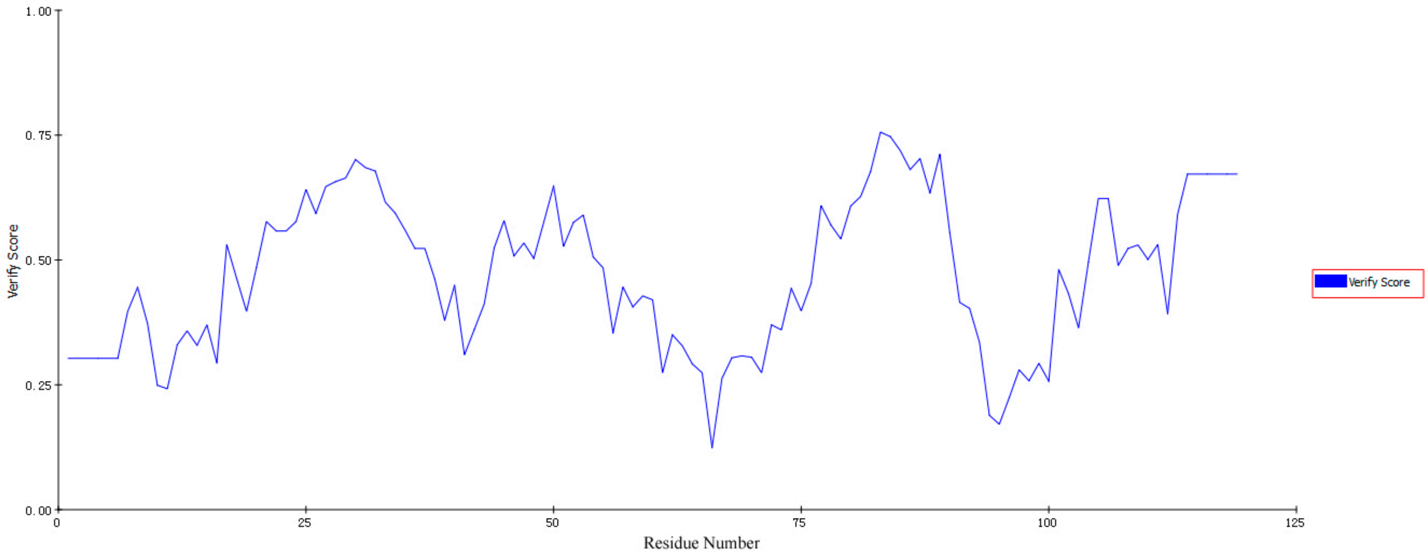

1.00
0.75
0.50
0.25
0.00
Verify Score
0
25
50
75
100
125
Residue Number
Verify Score

Figure S4

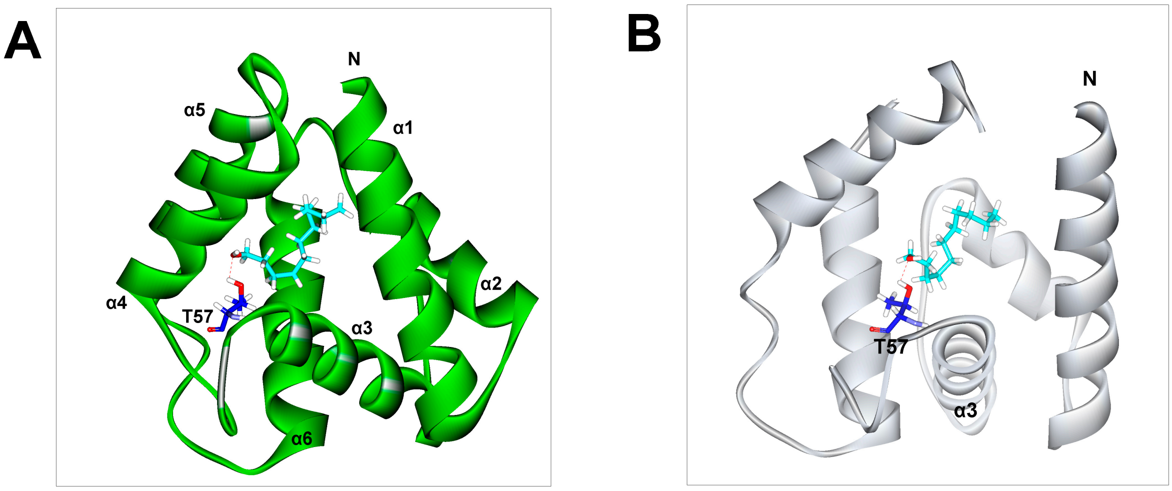

Figure S5.

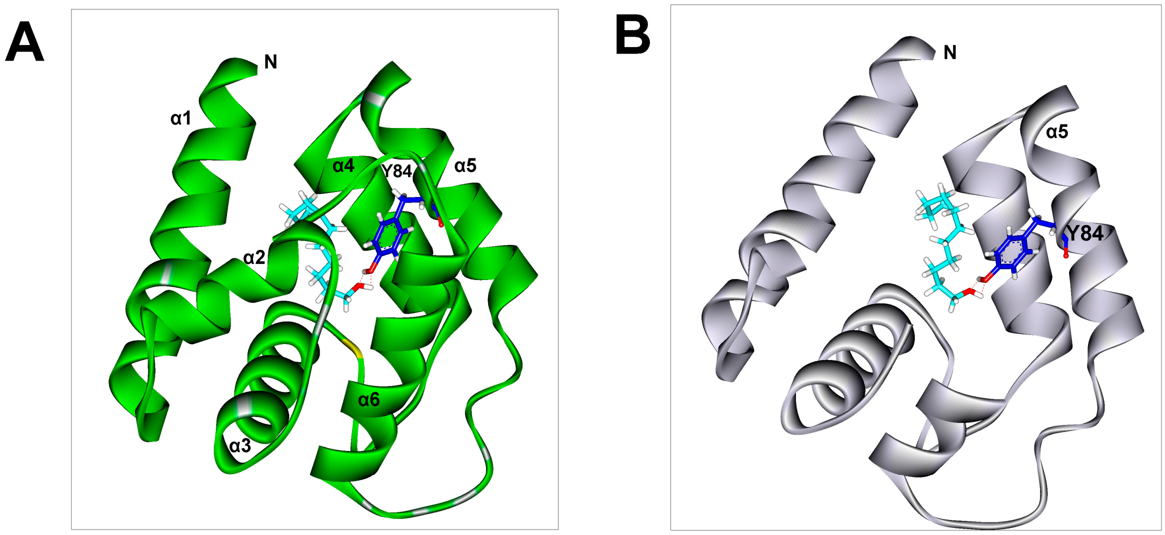

Figure S6

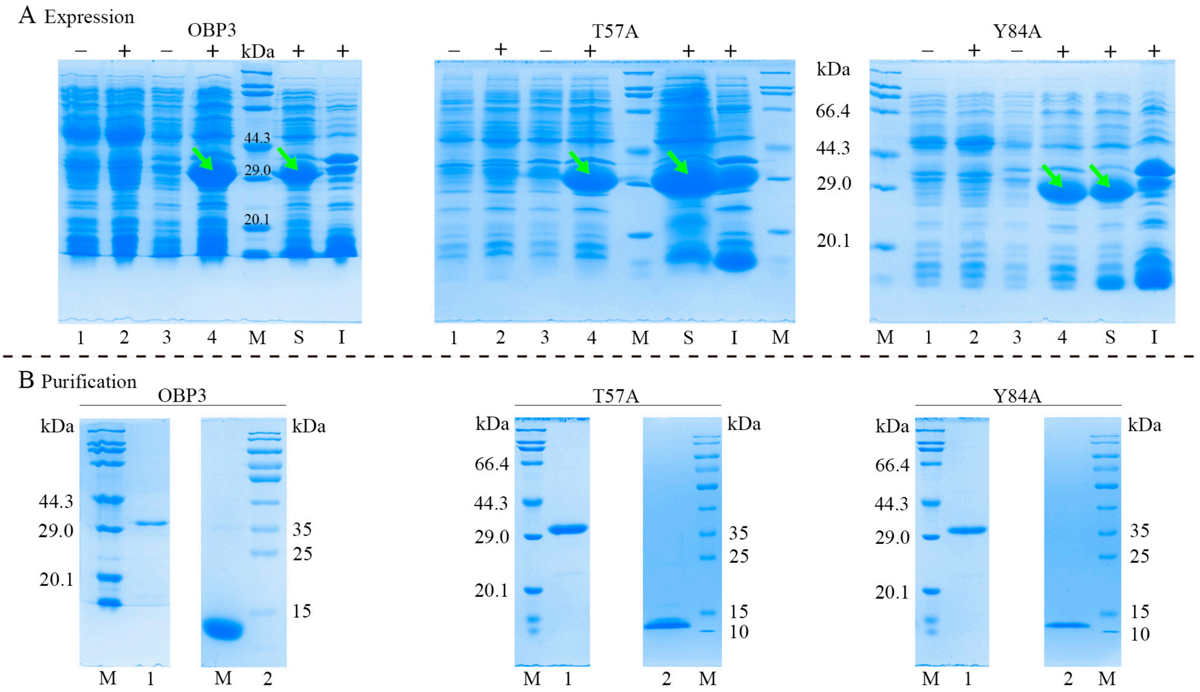
A Expression
OBP3
– + – + kDa + +
44.3
29.0
20.1
1 2 3 4 M S I
T57A
– + – + + +
1 2 3 4 M S I M
Y84A
kDa
66.4
44.3
29.0
20.1
– + – + + +
M 1 2 3 4 S I
B Purification
OBP3
kDa
44.3
29.0
20.1
M 1
kDa
35
25
15
M 2
T57A
kDa
66.4
44.3
29.0
20.1
M 1
kDa
35
25
15
10
2 M
Y84A
kDa
66.4
44.3
29.0
20.1
M 1
kDa
35
25
15
10
2 M

Figure S7

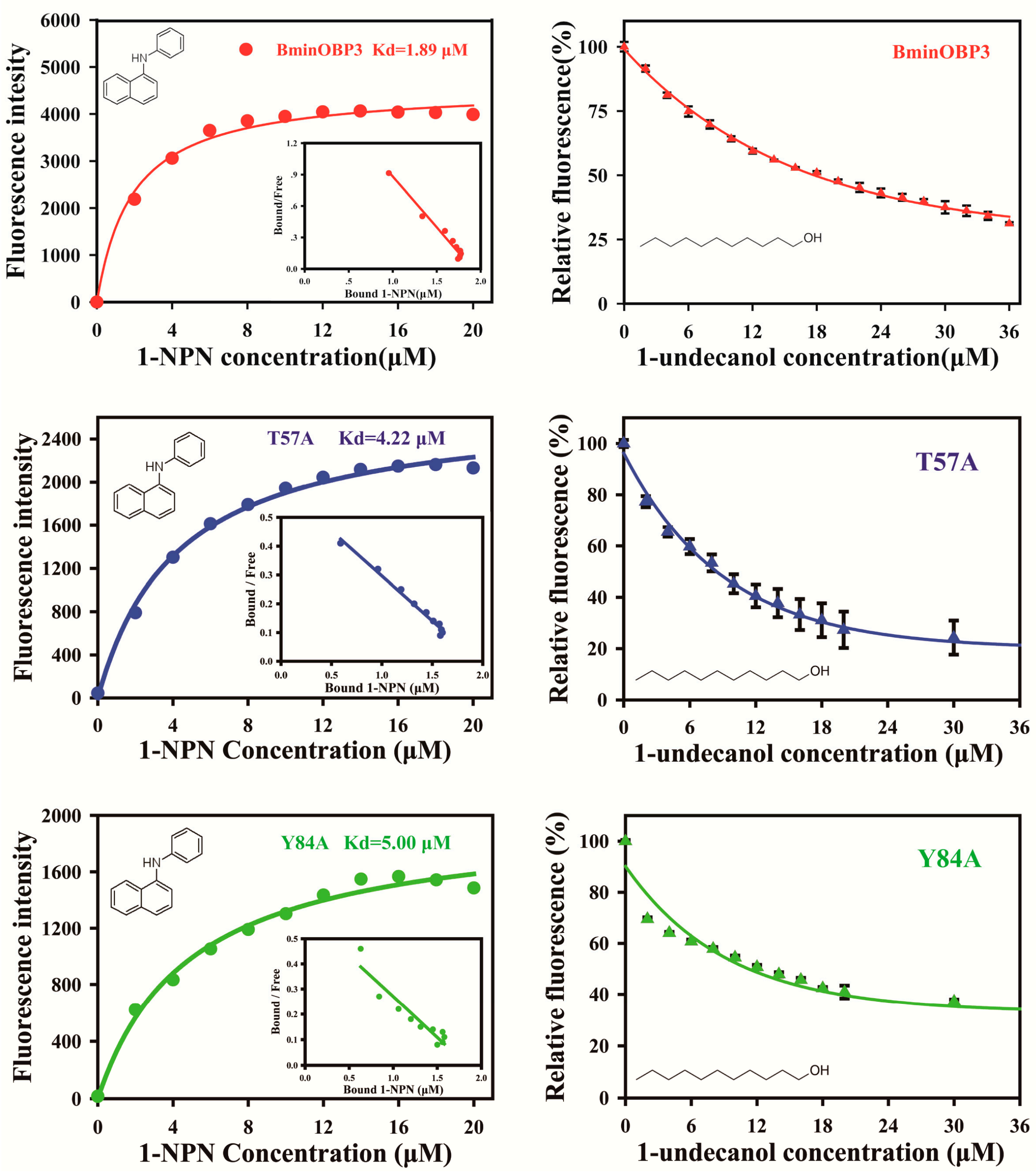
BminOBP3 Kd=1.89 μM
Fluorescence intesity
1-NPN concentration(μM)
Bound/Free
Bound 1-NPN(μM)
BminOBP3
Relative fluorescence(%)
1-undecanol concentration(μM)
T57A Kd=4.22 μM
Fluorescence intensity
1-NPN Concentration (μM)
Bound / Free
Bound 1-NPN (μM)
T57A
Relative fluorescence (%)
1-undecanol concentration (μM)
Y84A Kd=5.00 μM
Fluorescence intensity
1-NPN Concentration (μM)
Bound / Free
Bound 1-NPN (μM)
Y84A
Relative fluorescence (%)
1-undecanol concentration (μM)
HN
OH

Figure S8

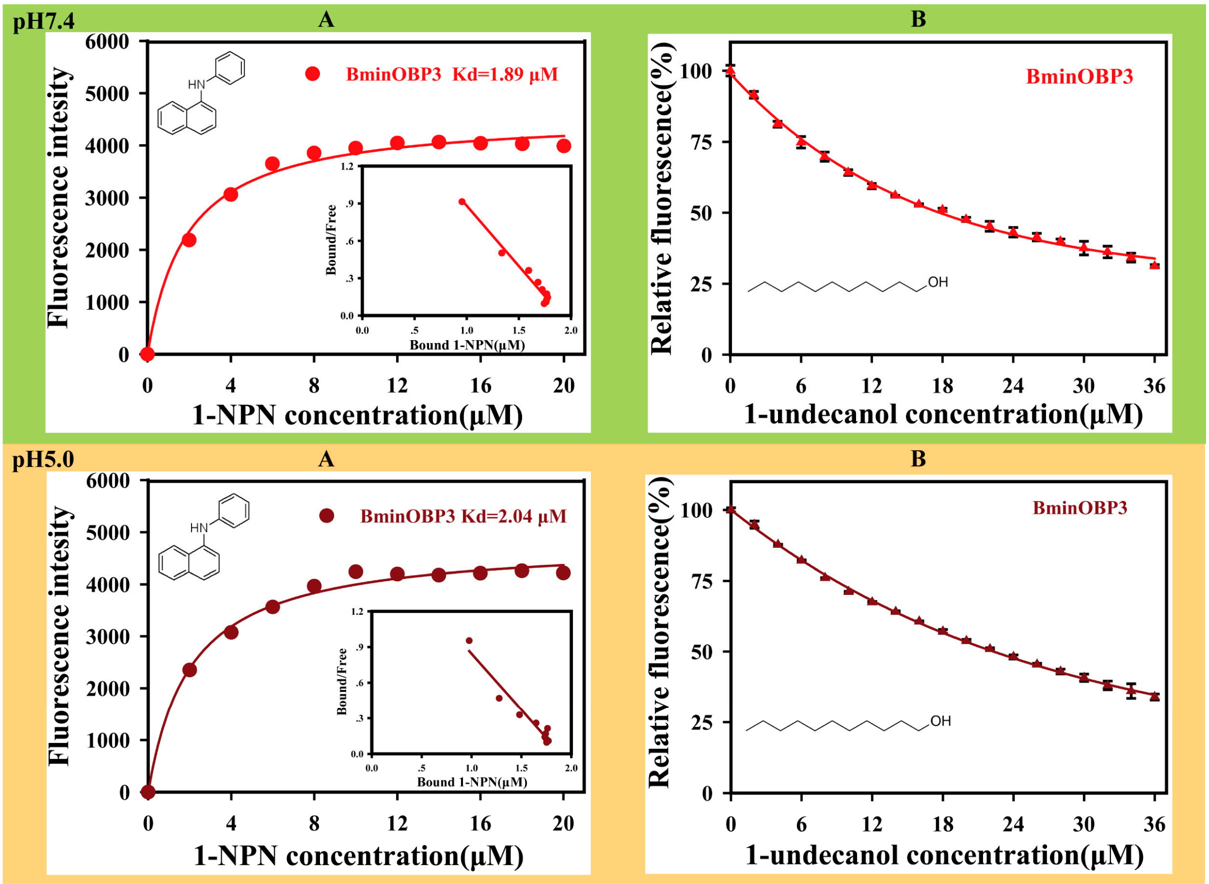
pH7.4
A
B
Fluorescence intesity
BminOBP3 Kd=1.89 μM
1-NPN concentration(μM)
Bound/Free
Bound 1-NPN(μM)
Relative fluorescence(%)
BminOBP3
1-undecanol concentration(μM)
OH
pH5.0
A
B
BminOBP3 Kd=2.04 μM
Fluorescence intesity
1-NPN concentration(μM)
Bound/Free
Bound 1-NPN(μM)
Relative fluorescence(%)
BminOBP3
1-undecanol concentration(μM)
OH

Table S1. Primers used for expressing BminOBP3 and its mutants BminOBP3-T57A and BminOBP3-Y84A

Purpose		Primer name	Primer sequence (5'-3')
Expression for the wild type BminOBP3		OBP3E-Forward	CGC**<u>GGATCC</u>**G$_{(1)}$GTGCTACGGAAGATC$_{(16)}$
		OBP3E-Reverse	CCC**<u>AAGCTT</u>**T$_{(369)}$TAAGGGAAAACAAAC$_{(354)}$
Expression for mutants	For the expression of the BminOBP3-T57A (threonine to alanine at position 57) mutant	T57A-Forward	A$_{(160)}$TGATGCAAGCAATGAAGAAA$_{(180)}$
		T57A-Reverse	T$_{(159)}$TCCATCACACAATTAATATAACA$_{(136)}$
	For the expression of the BminOBP3-Y84A (tyrosine to alanine at position 84) mutant	Y84A-Forward	A$_{(241)}$AGGATGATGCCCGCAACG$_{(259)}$
		Y84A-Reverse	G$_{(240)}$TAACTGTCCGGCATCAGCAAATC$_{(217)}$

The bold underlined letters in the primers indicate the restriction site for the enzymes *Bam*H I (forward primers) and *Han*d III (reverse primers). The numbers marked after the base in each primer indicate the positions of these bases on the nucleotide sequence. The red GCA, GCC showed the amino acid substitution: T57(ACA)-A(GCA), Y84(TAC)-A(GCC).

Table S2. Binding affinities of BminOBP3, T57A and Y84A to undecanol in fluorescence competitive binding assays.

Proteins	Undecanol	
	IC_{50} (μM)	K_i (μM)
BminOBP3	17.72±0.78	11.59±0.51 a
T57A	8.44±0.62	6.82±0.50 b
Y84A	11.02±0.49	9.18±0.40 c

Ki (inhibition constant) was calculated from the corresponding IC50 (the concentration of ligand when 1-NPN was replaced by half). BminOBP3: the wild type protein; T57A: BminOBP3-T57A (threonine to alanine at position 57) mutant; Y84A: BminOBP3-Y84A (tyrosine to alanine at position 84) mutant. K_i values within a column followed by different letter are significantly different [mean ± SE, ANOVA followed by Tukey's HSD test, $p < 0.05$]

Table S3. Binding affinities of BminOBP3 to 1-undecanol in fluorescence competitive binding assays at pH7.4 and pH5.0

Proteins	BminOBP3 to 1-undecanol	
	IC_{50} (μmol/L)	K_i (μmol/L)
pH7.4	17.72±0.78	11.59±0.51a
pH5.0	22.22±0.14	14.91±0.10 b

K_i (inhibition constant) was calculated from the corresponding IC_{50} (the concentration of ligand when 1-NPN was replaced by half); BminOBP3: the wild-type protein; T57A: BminOBP3-T57A (threonine to alanine at position 57) mutant; Y84A: BminOBP3-Y84A (tyrosine to alanine at position 84) mutant. K_i values within a column followed by different letter are significantly different [mean ± SE, Student's *t* test, $p < 0.05$]

Table S4 The source of 13 ligands used in the study reported by Chen et al. 2021

Compounds	CAS No.	Formula	Purity (%)	Source
Valeraldehyde	110-62-3	$C_5H_{10}O$	>97	Sigma
Methyl phenylacetate	101-41-7	$C_9H_{10}O_2$	>99	TCI
Butyl acetate	123-86-4	$C_6H_{12}O_2$	>99.5	Sigma
(R)-(+)-Limonene	5989-27-5	$C_{10}H_{16}$	>97	Sigma
Linalool	78-70-6	$C_{10}H_{18}O$	>97	Sigma
Isobutyraldehyde	78-84-2	C_4H_8O	>98	Sigma
Acetoin	513-86-0	$C_4H_8O_2$	>96	Sigma
(1s)-(-)-α-Pinene	7785-26-4	$C_{10}H_{16}$	>96	TCI
Benzyl alcohol	100-51-6	C_7H_8O	>99	TCI
Methyl salicylate	119-36-8	$C_8H_8O_3$	>99	TCI
Benzaldehyde	100-52-7	C_7H_6O	>99.5	aladdin
Undecanol	112-42-5	$C_{11}H_{24}O$	>99	TCI
1-Octanol	111-87-5	$C_8H_{18}O$	>99	TCI
